# Economic burden of Huntington’s disease in Peru

**DOI:** 10.1186/s12913-019-4806-6

**Published:** 2019-12-30

**Authors:** Gustavo Silva-Paredes, Rosa M. Urbanos-Garrido, Miguel Inca-Martinez, Danielle Rabinowitz, Mario R. Cornejo-Olivas

**Affiliations:** 1 Neurogenetics Research Center, Instituto Nacional de Ciencias Neurológicas, 1271 Ancash St, 15003 Lima, Peru; 2 School of Economics, University Complutense of Madrid, Madrid, Spain; 3 Lerner Research Institute, Genomic Medicine, Cleveland Clinic Foundation, Cleveland, OH USA; 4 Harvard Medical School, Boston, USA; 5 Center for Global Health, Universidad Peruana Cayetano Heredia, Lima, Peru

**Keywords:** Economic burden, Cost of illness, Direct costs, Indirect costs, Huntington’s disease

## Abstract

**Background:**

Huntington’s disease (HD) is a devastating and fatal neurodegenerative disorder that leads to progressive disability, and over time to total dependence. The economic impact of HD on patients living in developing countries like Peru is still unknown. This study aims to estimate the economic burden by estimating direct and indirect costs of Huntington’s disease in Peru, as well as the proportion of direct costs borne by patients and their families.

**Methods:**

Disease-cost cross-sectional study where 97 participants and their primary caregivers were interviewed using a common questionnaire. Prevalence and human capital approaches were used to estimate direct and indirect costs, respectively.

**Results:**

The average annual cost of HD reached USD 8120 per patient in 2015. Direct non-healthcare costs represented 78.3% of total cost, indirect costs 14.4% and direct healthcare costs the remaining 7.3%. The mean cost of HD increased with the degree of patient dependency: from USD 6572 for Barthel 4 & 5 (slight dependency and total independency, respectively) to USD 23,251 for Barthel 1 (total dependency). Direct costs were primarily financed by patients and their families.

**Conclusions:**

The estimated annual cost of HD for Peruvian society reached USD 1.2 million in 2015. The cost impact of HD on patients and their families is very high, becoming catastrophic for most dependent patients, and thus making it essential to prioritize full coverage by the State.

## Background

Huntington’s disease (HD) is a monogenetic, neurodegenerative disorder that leads to progressive disability and eventually to full dependence [1]. Age at onset spans from early childhood to senescence, although typical HD onset occurs in middle-aged patients [2]. HD symptoms are characterized by the triad of choreic movements, cognitive impairment, and psychiatric symptoms [3]. A variety of non-neurological clinical features are also associated, which therefore requires multidisciplinary and integrated clinical care [4] in order to improve quality of life and to reduce premature mortality [5].

The worldwide HD prevalence is estimated at 2.71 per 100,000 inhabitants [6], with wide variations among different regions [7]. There is a significant HD population in Latin America, mostly concentrated in Venezuela, Colombia, Peru and Brazil [8]. In Peru, there have been country-wide reports of HD, although the majority of patients are located in the capital city of Lima and in the Valley of Cañete (a HD hotspot with an estimated prevalence of 40 cases per 100,000 inhabitants) [9, 10]. According to a 2014 annual report, there have been at least 100 HD families diagnosed and currently receiving follow-up at the Neurogenetics Research Center at the *Instituto Nacional de Ciencias Neurológicas* (NRC-INCN), which is the only existing Peruvian center providing genetic testing for HD [11].

Despite the high prevalence of HD in Peru, public healthcare funding for HD patients is not a priority. In 2013, the Peruvian government promulgated a law declaring healthcare for rare disorders of high priority [12]; subsequently, the Ministry of Health of Peru (MINSA) developed a prioritized list of 399 rare diseases, with the recommendation to provide extra funding only to the top 8 [13]. HD ranks in the 297th position on the MINSA list, which means that it is not receiving this additional financial support.

Roughly 80% of the Peruvian population has health insurance of any kind [14]. The health care system comprises five subsystems [15, 16]: a) the MINSA subsystem, that provides health services to the poor and extremely poor (51% of the total population), although the full range of healthcare services (medical appointments, hospitalization, diagnostic tests and drugs) are only fully subsidized for SIS (integral health insurance) affiliated patients; thus, out of pocket payments are required for non-SIS affiliated patients; b) the Social Security Health subsystem (ESSALUD), which provides health services to the salaried and their dependents (26.7% of the population), run by the Ministry of Labor; c) the Armed Forces healthcare subsystem, run by the Ministry of Defense; d) the Police Forces healthcare subsystem, provided by the Home Office and e) a private healthcare subsystem covering approximately 1.8% of the population. Each subsystem consists of an independent legal framework, health coverage benefits, a technological infrastructure, and a network of healthcare centers and hospitals. Even for those having governmental insurance, cumbersome reference paperwork for insurance approval stands to hinder patients from receiving full coverage for many healthcare services. In Peru, technically there are no copayments in public healthcare. Even those drugs and medical devices which are directly provided by health care centers are for free, although if the patient has to purchase them in the pharmacy, he/she must pay the whole price.

Research regarding HD costs is very scarce [17, 18]. Furthermore, in Latin America and more specifically in Peru, there has been no research aimed at quantifying HD costs or defining the disease’s economic burden on afflicted patients. Our study aims to estimate direct and indirect costs of HD in Peru from a societal perspective, examining the economic impacts on both the healthcare system and on patients, and also on the economy as a whole, since we also compute productivity losses due to HD. To our knowledge, this study is the first to examine the economic burden of HD in Peru. It is our hope that it will contribute to fully understand the economic consequences of this disease in an effort to highlight the need for HD to become more of a priority for the Peruvian health care system.

## Methods

### Participants and materials

A standardized questionnaire (see additional file 1) was completed by 97 HD patients (or by his/her primary caregiver, when necessary based on level of dependence) who were receiving care at the neurogenetics clinic of the NRC-INCN, a tertiary healthcare institution and national referral center for HD located in Lima, Peru. We used the Epidat 4.0 software to determine the sample size, with a 95% confidence level. Every selected patient agreed to participate in the survey. Individual interviews were conducted from March 2014 to November 2015. All patients included in the study were adults (over 18 years) with confirmed molecular diagnosis of HD who had regular follow-up visits at the NRC-INCN for at least six months and who signed informed consent for study participation. IRB approval for the study was obtained at INCN. Each participant recruited for this study was classified into one of five groups based on disability status using the Barthel Index (ranging from 0 to 100), which assesses functional independence in ten basic activities of daily living and allows for categorization of patients from fully dependent (Barthel 0–20) to fully independent (Barthel 100) [19]. For this study, the Barthel index was divided into five categories: score < 20 = total dependency (labeled as Barthel 1); score 20–35 = severe dependency (Barthel 2); score 40–55 = moderate dependency (Barthel 3); score 60–99 = slight dependency (Barthel 4); and score 100 = total independency (Barthel 5) [19].

The patient questionnaire consisted of the following sections: 1) socio-demographic data; 2) use of health care services (including primary, secondary and tertiary services); 3) use of non-healthcare services (including informal long-term care, as well as transportation services to and from health care facilities); and 4) labor status and work limitations produced by the disease. The questionnaire surveyed costs and intensity of use of services and facilities over a six-month period. A one-month period was used for prescribed drugs, and a one-year period was considered in the questionnaire for hospitalizations. The period duration over which data were retrospectively collected is variable and based on frequency of use of different services, from most frequent (drugs) to least frequent (hospitalization), in order to facilitate patient recall. Information regarding who financed the service (i.e. health insurance or the patient him/herself through out-of-pocket payments) was also included.

The caregiver’s questionnaire (additional file 2) included socio-demographic data and labour status about the primary caregiver, and also detailed information about time spent by any caregiver in assisting in basic and instrumental activities of daily living for the given patient.

Both questionnaires were modified after preliminary analysis of a sample of 5 HD patients (pilot session). Edits included rephrasing of sentences for better understanding and adding more details to answers, including clarifying types of transportation and participants’ occupations. Interviews of the pilot session were excluded from the final analysis.

### Costing methodology

#### Healthcare direct costs

We used the prevalence approach for direct healthcare cost estimation [20]. Therefore, we calculated the costs generated by all existing cases in the reference year. A bottom-up costing approach was used to estimate the total and average annual costs which, following common practice in Peruvian economic reports, were expressed in US Dollars (the exchange rate used was 3.3 PEN = 1 USD). Healthcare services retrieved through the interviews included: follow-up appointments (both with specialists and primary care physicians), hospitalizations, complementary lab tests and neuroimaging, prescribed drugs, and use of medical devices (diapers, wheelchairs, dressings and bandages, articulable beds, etc.). No formal long-term care was reported by the interviewed individuals. Specific costs covered by health insurance were confirmed by checking the official price registry for the reference year period; by contrast, information on costs covered by patients individually were obtained directly from interviews. Costs of drugs were also estimated based on the MINSA price registry when supplied by the NRC-INCN [21], and from the National Observatory of Drugs (*Observatorio Peruano de Productos Farmacéuticos*) when they were not available at the INCN and had to be acquired by the patient in a pharmacy [22].

In order to provide yearly cost estimates, the cost of drugs and of use of healthcare services (except for hospitalizations) were multiplied by 12 and by 2, respectively. The year 2015 was used as the reference year.

#### Non-healthcare direct costs

Non-healthcare costs comprised transportation costs to health care facilities (and back home), as well as informal long-term caregiving costs. Information about transportation costs was obtained directly from patients. As the reference period was also in this case the six months prior to the interview, transportation costs were multiplied by 2 in order to compute annual costs.

Informal caregivers, usually family members or close friends, are those individuals who perform activities related to taking care of patients and who are usually not compensated for doing so [23]. Information regarding time spent on caregiving activities by any person was obtained directly from the caregiver’s questionnaire. Caregiving activities included: a) helping with basic activities of daily living (ADL) such as dressing, feeding, bathing, using the bathroom or ambulating; and also b) helping with instrumental activities of daily living (IADL) such as preparing meals, managing medications or transportation and shopping, among others. Data about time spent in basic activities were collected for a one-day period, whilst time spent in assisting in instrumental activities was referred to a one-week period. When the caregiver reported more than 16 hous a day, the number of daily hours was capped at 16 (implicitly considering 8 h of sleep time), by reducing the reported time spent on surveilling and supervising the patient.

A substitution method, also called “Proxy Good” method, was used to estimate informal care costs. This is a revealed preference method which values caregiving time spent considering a close substitute at the labour market. In other words, this technique values the care provided taking into account how much it would cost if informal caregivers would disappear and, consequently, they had to be replaced at the labour market by a close substitute [24, 25]. An approximate caregiving cost of USD 2.57/h was assigned by using data on the average market price of formal caregiving offered by four top ranked private companies, which was retrieved by web search. This price was multiplied by the estimated annual number of hours spent in caregiving.

#### Indirect costs

The questionnaire included information about the subjects’ labour status (working, unemployed, retired, student, housewife, temporary or permanent incapacity to work), but it also specifically includes questions about labour problems due to the disease: number of lost working hours and days in the latest year, whether the subject was forced to retire or quit the job and at what age, or even whether he/she never was able to work because of HD.

Indirect costs are those associated with temporary or permanent changes in work capacity/status for an HD patient as a result of his/her disease burden. We used the human capital approach for indirect cost estimation [26]. Thus, in our calculations we considered that one day off from work was equivalent to the corresponding one-day salary in the patient’s most recent job.

## Results

### Descriptive statistics

Descriptive statistics of the sample are shown in Table 1. Out of the 97 patients, 64% were female, 62.8% were married and about two-thirds had at least one child. The mean age at onset in our population was 42 years, with 75% of cases with age at onset over 30 years, which About one third of the patients held a post-secondary school degree. Only 17.5% of the individuals were still working and, among them, only 23% reported a formal job, which is consistent with the very high level of informal jobs in Peru (73.2% in 2015, according to the Peruvian Statistical Office) [27]. The birthplace distribution in our sample showed that HD participants were born in 15 of the 25 geographic regions of Peru, suggesting a broad distribution of cases within the country. However, most patients (68%) reported official residence in Lima. Only a reduced proportion of people affected by HD showed severe or total dependency (5.2%). Nevertheless, a majority of patients (68%) received help from a caregiver, who was always a close relative. For this particular subsample, the average daily hours of care ranged from 5 for Barthel 5 to 18.3 for Barthel 1. About 67% of patients were covered by some public insurance scheme, mostly that provided by the Ministry of Health and Social Security.

**Table 1:** Socio-demographics features of the sample

Characteristics	% or Mean ± SD (*n* = 97)
Female	63.9
Age	48.7 ± 13.6
Marital status	
Married/cohabiting	62.9%
Single	26.8%
Divorced/Separated	6.2%
Widowed	4.1%
Educational level	
Illiterate or incomplete primary school	6.2%
Complete primary school	10.3%
Incomplete secondary school	13.4%
Complete secondary school	36.1%
University studies	34.0%
Labor status	
Employed	17.5%
Unemployed	12.4%
Student	1.0%
Retired	8.2%
Disabled	37.2%
Homemaker	23.7%
Region of residence in Peru	
Lima (capital city)	68.0%
Callao	10.3%
Junín	7.2%
Ica	4.1%
Ayacucho	3.1%
Others^a^	7.3%
Barthel Index	
Barthel 1 (total dependency)	3.1%
Barthel 2 (severe dependency)	2.1%
Barthel 3 (moderate dependency)	9.3%
Barthel 4 (slight dependency)	50.5%
Barthel 5 (total independency)	35.0%
Caregiver assistance	68.0%
Daily care hours (conditioned to receive care)	
Barthel 1	18.3 ± 7.0
Barthel 2	17 ± 1.4
Barthel 3	13.4 ± 3.7
Barthel 4	9.8 ± 6.5
Barthel 5	5 ± 4.5
Type of health insurance	
Uninsured	32.0%
SIS	38.1%
ESSALUD	25.8%
Police & Armed Forces	3.1%
Private insurance	1.0%

^a^ Including 10 out of 25 regions (Arequipa, Apurímac, Huancayo, Ancash, Cajamarca, Cerro de Pasco, Ucayali, Huánuco, Cusco, La Libertad) in Peru

### Cost estimations

Due to the small size of our sample, cost estimations are provided after grouping the original five categories of Barthel into the following three: Barthel 1, Barthel 2 & 3 and Barthel 4 & 5. The annual average cost of HD was USD 8120, ranging from 6572 USD to 23,251 USD for patients classified as Barthel 4 & 5 and Barthel 1, respectively (Table 2). Non-healthcare direct costs represented the highest percentage of total cost for HD for all Barthel categories (78.3% on average), due to the weight of informal care. Indirect costs (wage losses) appeared as the second most important cost category (14.4% of total costs), and its weight tended to decrease as dependency increased. Finally, direct healthcare costs represented a residual proportion of the total (7.3% on average) except for the most disabled patients, for whom they accounted for 20.4%.

**Table 2:** Annual costs per patient with HD in Peru (USD, year 2015)

	Total (*n* = 97)		Barthel 1 (*n* = 3)		Barthel 2 & 3 (*n* = 11)		Barthel 4 & 5 (*n* = 83)	
	Per capita USD (SD)	%	Per capita USD (SD)	%	Per capita USD (SD)	%	Per capita USD (SD)	%
**Healthcare direct costs**	**590 (1075.2)**	**7.3%**	**4755 (2267.5)**	**20.4%**	**578 (605.4)**	**3.7%**	**441 (725.4)**	**6.7%**
Medicines	389 (952.4)	4.8%	3013 (3389.9)	13.0%	469 (622.8)	3.0%	284 (685.9)	4.3%
Ancillary services	44 (87.2)	0.6%	255 (220.9)	1.1%	26 (65.7)	0.2%	38 (74.1)	0.6%
Specialist visits	76 (174.9)	0.9%	333 (440.3)	1.4%	17 (7.5)	0.1%	75 (167.8)	1.1%
Primary care	9 (35.0)	0.1%	75 (82.7)	0.3%	14 (27.4)	0.1%	6 (31.6)	0.1%
Inpatient care	2 (24.6)	0.0%	81 (140.0)	0.3%	0	0.0%	0	0.0%
Medical devices	70 (266.5)	0.9%	998 (917.9)	4.3%	52 (172.9)	0.3%	38 (162.5)	0.6%
**Non-healthcare direct costs**	**6361 (6717.9)**	**78.3%**	**17,405 (6422.2)**	**74.9%**	**13,281 (3371.2)**	**84.7%**	**5044 (6148.4)**	**76.7%**
Informal caregivers	6161 (6629.1)	75.8%	17,144 (6590.9)	73.8%	13,197 (3413.3)	84.2%	4831 (6010.3)	73.5%
Transportation	200 (475.4)	2.5%	261 (279.5)	1.1%	84 (124.3)	0.5%	213 (508.6)	3.2%
**Indirect costs**	**1170 (2160.765)**	**14.4%**	**1091 (1889.5)**	**4.7%**	**1815 (1834.9)**	**11.6%**	**1087 (2214.8)**	**16.6%**
**Total costs**	**8121 (7533.699)**	**100.0%**	**23,251 (5739.1)**	**100.0%**	**15,674 (4383.7)**	**100.0%**	**6572 (6696.6)**	**100.0%**

*SD* Standard Deviation.

^a^*USD* (currency exchange rate 1 *USD* = 3.3 PEN)

**Fig. 1:**
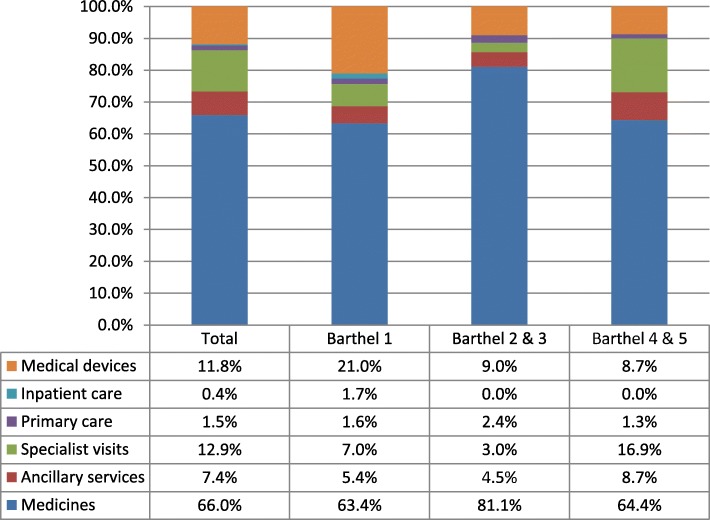
Components of direct healthcare costs for HD in Peru (2015)

The direct healthcare costs distribution (Fig. 1) showed that medicines represented the largest percentage (about 66% on average). For those patients with total dependency (Barthel 1), the proportion of healthcare costs corresponding to medical devices (diapers, wheelchairs, bandages, articulable beds, etc.) was also remarkable. Although specialist visits were the second component in the ranking of direct healthcare costs, its relative weight was quite low (12.9%). Most of these visits corresponded to neurology services (97%), though we found a minor proportion of visits to psychiatrists (1%) or genetic advisors (2%). This kind of medical attention is relatively concentrated within the patient population at earlier stages of HD. Hospitalizations were required only for the Barthel 1 group, with all indications for hospitalization associated with disease complications.

It is worth noting that inpatient care and medical devices were entirely financed by patients and their families out-of-pocket. For the rest of the healthcare costs, the average proportion of cost financed by the insurance scheme was meager and ranged from 0.5% for drugs to 9.4% for ancillary services. Even for medical visits, these percentages only reached 2.1 and 6.3% for general practitioners and specialists, respectively. As a consequence, the proportion of total medical costs paid out-of-pocket was 98.2%.

Regarding non-healthcare direct costs, transportation in all cases represented only a minor proportion of the total costs (from 0.5 to 3.2%, depending on the Barthel index) (Table 2), and was entirely financed by patients. Moreover, costs of informal caregiving showed a clear increase with the degree of dependency, as expected. Table 3 provides the caregiving time distribution according to the Barthel index, by distinguishing between basic and instrumental activities of daily living. Table 4 in Appendix provides full information about the distribution of time among activities, caregivers and Barthel categories.

**Table 3:** Distribution of informal caregiving by Barthel index (per capita daily hour average)

	Basic ADL		IADL		Surveillance & supervision		TOTAL	
	Primary caregiver	Secondary caregiver	Primary caregiver	Secondary caregiver	Primary caregiver	Secondary caregiver	Primary caregiver	Secondary caregiver
Barthel 1	4.2	2.6	7.2	1.7	2.6	0	14	4.3
Barthel 2 & 3	5.3	0.4	4.8	0.5	2.3	0.8	12.3	1.7
Barthel 4 & 5	0.5	0.2	2.2	0.5	1.4	0.5	4.0	1.1
TOTAL	1.1	0.3	2.6	0.5	1.6	0.5	5.3	1.3

According to our results, daily care hours ranged from 5.5 for less dependent patients to more than 18 for patients with total dependency, and were relatively concentrated on IADL (Table 3). Only for those patients with Barthel 4 & 5 the number of hours devoted to surveillance and supervision exceeded those employed in helping with basic ADL (Table 4 in Appendix).

Regarding indirect costs, wage losses were concentrated on disabled and employed patients, since those categorized as unemployed or retired reported that his/her labour status was not related to HD. According to our data, only patients totally independent or with slight dependency could work. For those working patients, the mean number of days off work in the latest year was 48.3, and the mean monthly salary reached USD 799.2. Moreover, only 2.1% of disabled could work some months during the latest year. The mean monthly salary for those patients, corresponding to their most recent job, reached USD 254.9.

Finally, we performed a means test in order to check whether the differences in large costs categories (direct health care, direct non-health care, indirect and total) were statistically significant in patients with total dependency and moderate-severe dependency vs. slight dependency-independent. Statistically significant differences were found for all cost categories (*p* = 0.000) except for indirect costs (*p* = 0.179).

## Discussion

The typical HD patient interviewed was an adult woman in a parental role who lived in the capital city of Lima. Higher prevalence of HD in Peruvian women was also found by Cornejo-Olivas et al. (2015) [10]. However, the relative higher frequency of female on the HD cohort we followed might do not reflect a real difference in HD population in Peru, and would be mostly related to demographic characteristics of outpatient HD population followed at the NRC, with less patients very ill and milder forms of HD. One of the characteristics of HD is that it appears when patients have already formed a family and have offspring [2, 3]. The mean age at onset in our population is consistent with classical reports among the majority of other worldwide HD cohorts previously reported on [28, 29]. Furthermore, the high percentage of our population reporting official residence in Lima is likely related to the fact that the NRC-INCN is the national referral center for HD in the public healthcare system [30].

According to our results, HD patients seemed to be affiliated with any health insurance in a lower proportion than the rest of Peruvian population. Therefore, they could be considered as a highly vulnerable group. Among patients working at the time of the interview, more than 76% declared their job as informal, which means that they did not have any work-associated benefits like health insurance coverage or a retirement pension. It may seem paradoxical that patients with Barthel 1, 2 or 3 in our cohort had, on average, similar salary losses than more independent patients (Barthel 4 or 5). This particular finding might be related to the fact that patients with higher disability might have lost their jobs a long time before the interview. Also, this result may be due to a sample bias, since the Barthel 1 category comprises a very small number of patients.

Up to 69% of the interviewed patients had a parental role and their HD status affected their ability to contribute to home expenses. The diagnosis of HD in one family member triggers many changes at home, often forcing another family member to take over the role of the person diagnosed with HD [31, 32]. Moreover, most patients need some kind of assistance to develop basic or instrumental activities of daily living, also leading to those closer relatives becoming the primary caregivers since the public social services network in Peru is virtually non-existent. Also, the cost of professional caregivers providing continued formal care is out of reach of most Peruvians and, in particular, of the patients in our sample, which explains that this kind of care was not reported. As was described in the results’ section, informal caregiving tends to increase with patients’ level of dependency. It is worth noting, however, that the total cost of informal caregiving services is higher in the Barthel 2 group (severe dependency) as compared with the Barthel 1 group (total dependency). This is related to the fact that most disabled HD patients are usually mute and confined to bed, thus requiring fewer hours of assistance in activities of daily living in comparison with those HD patients who are still walking.

The high percentage of informal caregiving costs shown in our study exceeds the one obtained in a United Kingdom study of a parallel HD cohort performed by Jones et al. [18], in which informal care of HD accounted for 65% of total costs. This fact may be related to the reduced size of the market of formal care in Peru. It also has to be highlightened that HD progressively generates motor disfunction (difficulties for walking, writing, dressing, feeding), cognitive (executive dysfunction, learning disability, memory loss, planning difficulties) and behavioral disturbances (apathy, hallucinations, depression, obsessive-compulsive behavior, suicidal behavior) [33]. Cost studies for Alzheimer’s Disease and other diseases causing severe dependency for activities of daily living also reported comparable results [23, 34].

By contrast, healthcare costs represented only 7.3% of total costs. The average cost reached USD 590 in 2015, almost twice the total health care expenditure per inhabitant in Peru in the same year (USD 314) [35]. As anticipated, healthcare costs increased in parallel with increases in patient dependency, in line with previous literature [17]. With regard to medical visits, most of our patients only reported follow-up visits with neurology/neurogenetics, contrasting with national and international HD guidelines. These guidelines recommend an integral healthcare strategy for HD patients that includes, in conjunction with symptomatic pharmacological therapies, regular visits to neurologists, geneticists, psychiatrists/psychologists, endocrinologists, gastroenterologists, nutritionists, rehabilitation specialists and general practitioners, among others [36, 37].

Almost all interviewed patients had to finance healthcare costs out-of-pocket, despite most of them technically being enrolled in some form of public health insurance. It is worth noting that INCN is the usual provider of healthcare services for HD patients, and that these services may only be provided for free to those citizens who are covered by the SIS (integral health insurance). Even so, patients are forced to deal with the bureaucracy of SIS, which requires extensive referral documentation and additional paperwork to be completed beforehand, and also leads to months-long waits for reimbursements. Since most patients opted to finance healthcare costs out-of-pocket and did not pursue SIS reimbursement given logistical constraints, it is reasonable to assume that they only could afford high priority drugs and medical visits. This fact could explain the large proportion of reported neurology follow-up visits versus visits to other physicians.

Our study has a number of limitations. Firstly, it is possible that a certain number of HD patients residing in provinces with challenging environments, such as the highlands or jungle, have limited access to molecular diagnosis due to geographical barriers, which would thus contribute to sample bias. Nevertheless, as was shown in the results’ section, there is a reasonable geographic spread in our sample. Secondly, since the use of health services depends mostly on the spending capacity of patients and their families rather than on optimal healthcare requirements, healthcare costs will be underestimated relative to what appropriate treatment would require. Thirdly, we cannot provide a sensitivity test for our estimations of informal care costs by using an opportunity cost method, since socio-demographic and labour status data were only collected for the main caregiver. Finally, external validity of our study may also be limited as our sample derives from a population registered in the NRC-INCN, which does not necessarily represent the entirety of the Peruvian HD population. However, since the NRC-INCN is the only national reference center for molecular diagnosis of HD in the public system, almost every Peruvian patient with a clinical suspicion for HD is referred to this center in order to determine a definitive diagnosis.

## Conclusions

As there is no national registry of HD prevalence in Peru, we cannot make an accurate calculation of societal cost of HD. However, we may estimate a low threshold by assuming that the national prevalence is in line with the minimal prevalence rate reported for Latin America (0.35 per 100,000 inhabitants, based on the community-based prevalence of HD in Venezuela) [7] and by taking into consideration that the estimated prevalence in the Valley of Cañete (a known HD focus) is 40 per 100,000 inhabitants [9]. Therefore, we can estimate that the total annual cost of HD for Peruvian society reached about USD 1.2 million in 2015, with direct healthcare costs accounting for around USD 85 thousand. This latter amount only represents 0.15% of the total budget allocated in 2015 to the Intangible Solidarity Health Fund (FISSAL), which is the public agency in charge of financing high cost and rare diseases. However, today HD is excluded from the list of the eight high priority rare diseases covered by the FISSAL.

According to our results, 98.2% of direct healthcare costs and 100% of direct non-healthcare costs are borne by patients and their families. Medical care costs represent, on average, 19.3% of the annual equivalent household income, but it dramatically increases with patients’ dependency degree (from 14.7% for Barthel 4 and 5 to 25.2% for Barthel 3, and 85.4% for most disabled patients --Barthel 1 and 2-). WHO defines as catastrophic those health expenditure “greater than or equal to 40% of a household’s non-subsistence income, i.e. income available after basic needs have been met” [38]. Therefore, we conclude that the impact of HD cost on patients and their families is high, and catastrophic for most dependent patients. Aside from the significant economic barriers, patients with HD also suffer from a lack of appropriate access to integral care due to the existence of geographical and administrative barriers, and also due to the limited supply of specialized medical services in the Peruvian healthcare system.

Additional federal funding for this rare disease would therefore significantly reduce the out-of-pocket payments for HD patients and allow them to receive the needed level of care for optimal disease management. Thus, it is our hope that defining the economic impact of HD on patients and their families will help pave the way for the development of new health policies that aim to more appropriately address the health and social needs of this vulnerable population.

### Supplementary information

**Additional file 1.** Patient questionnaire. Questionnaire completed by the HD patients or by his/her primary caregiver, when necessary.

**Additional file 2.** Main caregiver questionnaire. Questionnaire completed by the main caregiver about caregiving activities.

## Data Availability

The database created and analyzed for this study are available from the corresponding author on reasonable request.

## Appendix

**Table 4:** Distribution of informal caregiving by Barthel index (per capita annual hour average)

	Barthel 1				Barthel 2				Barthel 3				Barthel 4				Barthel 5			
	PC	SC	Total	%	PC	SC	Total	%	PC	SC	Total	%	PC	SC	Total	%	PC	SC	Total	%
Personal hygiene & dressing	321	364	685	10.3%	546	0	546	8.8%	506	105	611	12.5%	49	26	76	3.0%	5	0	5	0.6%
Bathing or showering	191	104	295	4.4%	61	0	61	1.0%	305	56	361	7.4%	44	12	56	2.2%	11	0	11	1.2%
Feeding	546	364	910	13.6%	364	0	364	5.9%	283	20	303	6.2%	64	11	75	2.9%	0	0	0	0.0%
Functional mobility	485	121	606	9.1%	2275	0	2275	36.7%	541	1	542	11.1%	106	44	150	5.9%	22	0	22	2.4%
TOTAL Basic ADL	1543	953	2496	37.4%	3246	0	3246	52.3%	1635	182	1817	37.1%	263	93	356	13.9%	38	0	38	4.2%
Preparing meals	768	364	1132	17.0%	455	0	455	7.3%	940	172	1112	22.7%	375	85	461	18.0%	197	11	208	22.9%
Doctor consultations & medical test	128	0	128	1.9%	18	0	18	0.3%	26	1	27	0.6%	42	5	47	1.8%	55	7	62	6.8%
Managing medication/nursery	708	243	950	14.2%	228	0	228	3.7%	95	12	107	2.2%	77	12	89	3.5%	32	24	56	6.2%
Housework	711	0	711	10.7%	520	0	520	8.4%	522	6	528	10.8%	247	42	289	11.3%	159	6	165	18.1%
Moving within the community	52	0	52	0.8%	5	0	5	0.1%	16	3	19	0.4%	40	14	54	2.1%	57	24	81	8.9%
Shopping	208	0	208	3.1%	137	0	137	2.2%	69	10	79	1.6%	64	15	79	3.1%	6	11	17	1.8%
Financial or administrative management	32	0	32	0.5%	0	0	0	0.0%	1	1	1	0.0%	3	0	3	0.1%	2	0	2	0.2%
Social or recreational activities	24	0	24	0.4%	0	0	0	0.0%	154	3	157	3.2%	66	44	110	4.3%	104	34	138	15.2%
TOTAL Instrumental ADL	2631	607	3237	48.5%	1363	0	1363	21.9%	1823	208	2031	41.5%	914	218	1132	44.3%	612	117	729	80.2%
Surveillance & supervision	938	0	938	14.1%	1233	364	1597	25.7%	749	300	1049	21.4%	796	270	1066	41.7%	99	42	141	15.6%
TOTAL	5111	1560	6671	100.0%	5842	364	6206	100.0%	4207	690	4897	100.0%	1973	581	2554	100.0%	749	159	908	100.0%

*PC* primary caregiver; *SC* secondary caregiver

## Abbreviations

ADL: Activities of daily living
ESSALUD: Social Security Health Subsystem
FISSAL: Intangible Solidarity Health Fund
HD: Huntington’s disease
MINSA: Ministry of Health of Peru
NRC-INCN: Neurogenetics Research Center -*Instituto Nacional de Ciencias Neurológicas*
SIS: Integral Health Insurance
USD: United States Dollars
