# Supplementary material for: Economic burden of Huntington’s disease in Peru
Source: BMC Health Serv Res. 2019 Dec 30;19:1017. doi: 10.1186/s12913-019-4806-6 (PMC6937635; doi:10.1186/s12913-019-4806-6)
Supplement: Supplementary file 1 — Additional file 1. Patient questionnaire. Questionnaire completed by the HD patients or by his/her primary caregiver, when necessary. [file 12913_2019_4806_MOESM1_ESM.docx]

Patient questionnaire

Patient code………………………………………………………………….Date…………………………

| 1. Sex | Male () Female () |
| --- | --- |
| 2. Marital status | Single () Married () Living with a partner () Divorced/Separated () Widowed () |
| 3. Birth date |  |
| 4. Place at birth | Department: Province: |
| 5. Place of residence | Department: Province: |
| 6. Age at the moment the disease was diagnosed |  |
| 7. Educational level | Illiterate () Primary education () Secondary education () Higher education()  Completed educational level () Uncompleted () |
| 8. Role played at the household | Father/mother () Husband/wife() Daughter/son() Other() |
| 9. Number of members of the household |  |
| 10. Number of members of the household affected by HD |  |
| 11. Total monthly income of the household (in Peruvian soles) |  |
| 12. Health insurance | Yes () Affiliation date: No() |
| 13. Type of insurance | SIS() ESSALUD() Armed Forces() Police Forces() Private insurance() Uninsured () |
| 14. Do you need the help of a caregiver to develop his/her daily activities? (personal hygiene, functional mobility, managing medication, etc.) | Yes()  No() (Go to question 17) |
| 15. Type of caregiving | Informal caregiver (not paid: relative, friend) (Go to question 17)  Professional (paid) caregiver |
| 16. If professional care is received | How many hours per week?  Who pays for the service?  How much is paid per hour? |
| 17. Labour status | Working () Activity () Wage ()  Unemployed (Go to question 20)  Retired (Go to question 19)  Student (Go to question 20)  Homemaker (Go to question 20)  Temporary incapacity to work (Go to question 20)  Permanent incapacity to work (Go to question 19)  Other situation (Go to question 20) |
| 18. Have you suffered any labour limitation in the last 12 months due to HD? | No (Go to question 20)  Yes (). In that case:  -I have been in medical break for _days  -I have been working _hours less per day for _ days  -I am working _hours less per day  -I work the same number of hours, but I have problems to perform at work  -Other problems:___ |

19. Job abandonment (only if the patient is retired or in a situation of permanent work disability)

-Yes ()

If yes, indicate how:

- I had to leave my job at the age of___
- I had to early retire at the age of___

-No ()

-I have never been able to work as a result of my illness ()

20. What medication have you been taking during the last month (due to your illness)?

| Drug | Is the cost covered by the IAFAS? | | | Monthly cost |
| --- | --- | --- | --- | --- |
|  | Yes | No | Partially (%) |  |
| Olanzapine |  |  |  |  |
| Risperidone |  |  |  |  |
| Tetrabenazine |  |  |  |  |
| Sulpiride |  |  |  |  |
| Haloperidol |  |  |  |  |
| Fluoxetine |  |  |  |  |
| Clonazepan |  |  |  |  |
| Sertraline |  |  |  |  |
| Quetiapine |  |  |  |  |
| Valproic acid |  |  |  |  |
| Chlorpromazine |  |  |  |  |
| Escitalopram |  |  |  |  |
| Alprazolam |  |  |  |  |
| Others (specify) |  |  |  |  |

21. What kind of diagnostic tests prescribed by a doctor have you undergone in the last 6 months (due to your illness)?

| Diagnostic tests in the last 6 months | Number | Is the cost covered by the IAFAS? | | | Unit cost per diagnostic test |
| --- | --- | --- | --- | --- | --- |
|  |  | Yes | No | Partially (%) |  |
| Genetic study |  |  |  |  |  |
| Blood test |  |  |  |  |  |
| Urine test |  |  |  |  |  |
| Brain tomography |  |  |  |  |  |
| MRI (magnetic resonance) |  |  |  |  |  |
| Others (specify) |  |  |  |  |  |

22. How many visits to the specialist did you make in the last 6 months (due to your illness)?

| Specialist | Number | Is the cost covered by the IAFAS? | | | Unit cost per visit |
| --- | --- | --- | --- | --- | --- |
|  |  | Yes | No | Partially (%) |  |
| Genetic counseling |  |  |  |  |  |
| Neurologist |  |  |  |  |  |
| Traumatologist |  |  |  |  |  |
| Endocrinologist |  |  |  |  |  |
| Gatroenterologist |  |  |  |  |  |
| Psychiatrist |  |  |  |  |  |
| Cardiologist |  |  |  |  |  |
| Pediatrician |  |  |  |  |  |
| Physiotherapist |  |  |  |  |  |
| Nutritionist |  |  |  |  |  |
| Psychologist |  |  |  |  |  |
| Neuropsychologist |  |  |  |  |  |
| Others (specify) |  |  |  |  |  |

23. How many visits to the GP/ER did you make in the last 6 months (due to your illness)?

| Visits | Number | Is the cost covered by the IAFAS? | | | Unit cost per visit |
| --- | --- | --- | --- | --- | --- |
|  |  | Yes | No | Partially (%) |  |
| GP at the primary health care center |  |  |  |  |  |
| Private doctor |  |  |  |  |  |
| Emergency room |  |  |  |  |  |

24. Inpatient care: have you spend at least one night in hospital in the last 12 months (due to your illness)?

-Yes (). How many times? __

-No (). Go to question 26.

| Times at hospital in the last 12 months | Is the cost covered by the IAFAS? | | | Unit cost per hospitalization |
| --- | --- | --- | --- | --- |
|  | Yes | No | Partially (%) |  |
| 1. |  |  |  |  |
| 2. |  |  |  |  |
| 3 |  |  |  |  |
| 4. |  |  |  |  |
| … |  |  |  |  |

25. What kind of barriers have you faced to access your health care?

- Absence of health insurance ()

- Partial coverage of health insurance ()

- Distance to the hospital ()

- Waiting list to get an appointment () Months___ Days___

- Transportation cost ()

- No economic resources ()

- Others (specify)_______

26. What kind of devices have you used in the last 6 months (due to your illness)?

| Devices | Number | Is the cost covered by the IAFAS? | | | Unit cost per device |
| --- | --- | --- | --- | --- | --- |
|  |  | Yes | No | Partially (%) |  |
| Articulated bed |  |  |  |  |  |
| Wheelchair |  |  |  |  |  |
| Walking stick |  |  |  |  |  |
| Splints |  |  |  |  |  |
| Dressings & bandages |  |  |  |  |  |
| Nasogastric tube |  |  |  |  |  |
| Food supplements |  |  |  |  |  |
| Diapers |  |  |  |  |  |
| Special shoes |  |  |  |  |  |
| Special clothes |  |  |  |  |  |
| Housing adaptation devices |  |  |  |  |  |
| Automobile adaptation devices |  |  |  |  |  |
| Others (specify) |  |  |  |  |  |

27. What kind of difficulties have you faced to get medical devices?

- No stock ()

-The product does not exist anymore ()

-It has to be imported from another country ()

-It is prescribed for other illness, but not for mine ()

-It is not covered by the health insurance and it is too costly for me ()

-Others (specify)___

28. How many times have you used the following transportation services in the last 6 months, for those transfers to the health care center, hospital, rehabilitation center, etc., related to your illness?

| Transportation services | Number of times | Is the cost covered by the IAFAS? | | | Unit cost per transport |
| --- | --- | --- | --- | --- | --- |
|  |  | Yes | No | Partially (%) |  |
| Private car |  |  |  |  |  |
| Public transport |  |  |  |  |  |
| Taxi |  |  |  |  |  |
| Inter-province transport |  |  |  |  |  |
| Ambulance |  |  |  |  |  |
| Plane |  |  |  |  |  |
| Others (specify) |  |  |  |  |  |

29. What kind of long-term care have you used in the last 6 months?

| Type of long-term care | Number | Is the cost covered by the IAFAS? | | | Unit cost per service |
| --- | --- | --- | --- | --- | --- |
|  |  | Yes | No | Partially (%) |  |
| Healthcare at home |  |  |  |  |  |
| Social assistance at home |  |  |  |  |  |
| Day care centers |  |  |  |  |  |
| Occupational centers |  |  |  |  |  |
| Telecare |  |  |  |  |  |
| Others (specify) |  |  |  |  |  |

30. Please, indicate your satisfaction with the healthcare received due to your illness in the following scale:

1 ( ) 2 ( ) 3 ( ) 4 ( ) 5 ( ) 6 ( ) 7 ( ) 8 ( ) 9 ( ) 10 ( )

Not at all satisfied Completely satisfied

31. Who has answered the questionnaire?

- The patient with HD

- The patient with HD assisted by a relative, caregiver, tutor, etc.

- A relative, caregiver, tutor…on behalf of the patient
