# Supplementary material for: Economic burden of Huntington’s disease in Peru
Source: BMC Health Serv Res. 2019 Dec 30;19:1017. doi: 10.1186/s12913-019-4806-6 (PMC6937635; doi:10.1186/s12913-019-4806-6)
Supplement: Supplementary file 2 — Additional file 2. Main caregiver questionnaire. Questionnaire completed by the main caregiver about caregiving activities. [file 12913_2019_4806_MOESM2_ESM.docx]

Main caregiver questionnaire

Patient code………………………………………………………………….Date…………………………

| 1. Sex | Male () Female () |
| --- | --- |
| 2. Marital status | Single () Married () Living with a partner () Divorced/Separated () Widowed () |
| 3. Birth date |  |
| 4. Place of residence | Department: Province: |
| 5. Relationship with the patient | Daughter/son () Mother/Father () Brother/sister () Daughter in law/son in law () Other (specify)__ |
| 6. For how long have you been caring the patient | Years____Months____ |
| 7. Educational level | Illiterate () Primary education () Secondary education () Higher education()  Completed educational level () Uncompleted () |
| 8. Labour status: | Working ()  Unemployed ()  Retired (Go to question 10)  Student (Go to question 11)  Homemaker (Go to question 11) |

9. Have you had any labour problem in the last 12 months due to your role as a caregiver?

- No ()

- Yes (). In that case, how did it affect your job?

- I requested___days of leave

- I worked ___less hours a day for ___days

- I am working ___less hours a day since___

- I work my regular workday but I have some problems keeping my schedule

- I don’t get a job compatible with my caregiver role

- Others (specify)

10. Did you have to retire early in order to take care of the patient?

- No ()

- Yes (). In that case, please indicate at what age did you retire:__

Regarding your role as the main caregiver

11. How much time do you approximately spend EVERY DAY in the following patient-related activities?

| Activity | Hours | Minutes |  |
| --- | --- | --- | --- |
| Personal hygiene & dressing |  |  | Per day |
| Bathing or showering |  |  | Per day |
| Feeding |  |  | Per day |
| Functional mobility |  |  | Per day |
| Preparing meals |  |  | Per day |
| Managing medication |  |  | Per day |
| Nursery |  |  | Per day |

12. How much time do you approximately spend WEEKLY in the following patient-related activities?

| Activity | Hours | Minutes |  |
| --- | --- | --- | --- |
| Doctor consultations & medical test |  |  | Per week |
| Housework |  |  | Per week |
| Moving within the community |  |  | Per week |
| Shopping |  |  | Per week |
| Financial or administrative management |  |  | Per week |
| Surveillance & supervision |  |  | Per week |

Regarding the role developed by other caregivers

13. How much time do they approximately spend EVERY DAY in the following patient-related activities?

| Activity | Hours | Minutes |  |
| --- | --- | --- | --- |
| Personal hygiene & dressing |  |  | Per day |
| Bathing or showering |  |  | Per day |
| Feeding |  |  | Per day |
| Functional mobility |  |  | Per day |
| Preparing meals |  |  | Per day |
| Managing medication |  |  | Per day |
| Nursery |  |  | Per day |

14. How much time do they approximately spend WEEKLY in the following patient-related activities?

| Activity | Hours | Minutes |  |
| --- | --- | --- | --- |
| Personal hygiene & dressing |  |  | Per day |
| Bathing or showering |  |  | Per day |
| Feeding |  |  | Per day |
| Functional mobility |  |  | Per day |
| Preparing meals |  |  | Per day |
| Managing medication |  |  | Per day |
| Nursery |  |  | Per day |
